# Interventional Radiology for Disease Management: A Narrative Review

**DOI:** 10.7759/cureus.48603

**Published:** 2023-11-10

**Authors:** Rasam Mashoufi, Ramin Mashoufi

**Affiliations:** 1 Innovative Medical Research Center, Faculty of Medicine, Mashhad Medical Science, Islamic Azad University, Mashhad, IRN; 2 Department of Radiology, Faculty of Medicine, Mashhad University of Medical Sciences, Mashhad, IRN

**Keywords:** magnetic resonance imaging, interventional oncology, imaging, disease management, interventional radiology

## Abstract

Interventional radiology (IR) is a branch of radiology that has helped many people who needed extensive surgery. In light of IR's significance in medicine, the main objective of this study was to perform a narrative review of IR uses in illness management. The results of this study were compiled based on the method of conducting review studies. To carry out this study, a systematic search was conducted in international databases, including Web of Science, ScienceDirect, Scopus, PubMed, and Google Scholar, in the time range of 2000-2023, using relevant keywords. According to the search and considering the necessary criteria to collect the results, 40 articles were selected, and the data was retrieved from them. This study found that IR is useful in obtaining tissue samples for further analysis. Imaging to guide a biopsy is also a widespread practice among interventional radiologists. Previous research has shown that the field of IR/interventional oncology undergoes rapid advancement through the continuous modification of techniques and the development of new treatment methods with minimal invasion. Based on the present study's findings, it can be concluded that IR is an alternative to surgery in some cases and, therefore, can reduce the high cost for patients. In addition, IR reduces the complications related to surgery and the hospitalisation time of patients.

## Introduction and background

Interventional radiology (IR) is a distinct subspecialty within the field of radiology, encompassing a range of modalities including fluoroscopy, computed tomography (CT) scanning, ultrasound (US), and magnetic resonance imaging (MRI). This discipline employs specialised techniques and clinical expertise to both diagnose and treat a diverse array of diseases [1]. In this therapeutic approach, the interventional radiologist and their team employ radiographic modalities to capture images of the specific organ of interest. These modalities serve as a guiding mechanism for directing the tools and equipment involved in the procedure towards the intended target organ or site. Subsequently, the treatment is executed [2]. Numerous ailments that previously necessitated extensive surgical procedures are now managed through the utilisation of IR techniques. The majority of IR treatments are conducted using small skin punctures rather than larger surgical incisions, indicating that these procedures are characterised by their minimally interventional nature [3].

IR encompasses two primary disciplines, namely, vascular IR and non-vascular IR [4]. The field of vascular IR is employed for the purpose of arterial stenosis and acute arterial occlusion. During the process of venous constriction, specialised springs are employed to alleviate vascular obstruction. These springs are directed by imaging instruments and are introduced into the affected region through the correct groin. Subsequently, the vessel undergoes dilation with an angioplasty balloon, followed by the placement of stents, which are specialised springs, along the stenosis [5]. During instances of abrupt occlusion of the arteries, the management of this issue involves the administration of anticoagulant medications via tiny catheters directly into the affected artery [6].

The use of medical imaging for cancer screening is a widely practised process in many countries. In situations where cancer is suspected, medical imaging is commonly adopted as the primary diagnosis method. Breast and lung cancer screening images are strongly advocated by many societal norms. In the last two decades, significant advancements have been achieved in the field of medical imaging, facilitating expedited and precise cancer diagnoses. Consequently, these advancements have resulted in improved prognoses and overall treatment outcomes for individuals afflicted with cancer [7].

The application of medical imaging modalities is crucial in promptly assessing the effectiveness of treatment and in the first detection and diagnosis of neoplastic disorders. CT and MRI are the prevailing cross-sectional imaging techniques commonly employed for the initial assessment or, in exceptional cases, for staging purposes. The issuance of treatment guidelines for different types of malignancies based on their anatomical location is carried out by the National Comprehensive Cancer Network (NCCN). The NCCN additionally provides guidance on the early detection, prevention, and reduction of cancer risk, taking into account the exact anatomical location from which the disease originates. The NCCN guidelines include evidence-based decision-making algorithms that can be utilised to treat about 97% of cancers [8,9]. Given the significance of IR within the realm of medical science, the primary objective of this study is to explore the use of IR in the management of diseases, drawing upon prior research and experiential knowledge in this domain.

## Review

Material and methods

Systematic searches in international databases like Web of Science, ScienceDirect, Scopus, PubMed, and Google Scholar were conducted from December 2000 to June 2023 to review and extract relevant articles and reports. Narrative review using Medical Subject Headings (MeSH) terms "Radiology", "Interventional Radiology", "Oncology", "Disease Management", "Treating Diseases", "Imaging", "Biopsy Under Imaging", "Ureteral Stenting", "Nephrostomy Under Imaging", "Biliary Interventions", "Chorionic Villus Sampling", "Amniocentesis Under Ultrasound Guidance", "Angiography Under Imaging", "Angioplasty", "Stenting in Vessels", "Application of Laser in Medicine", "Radiofrequency", "Embolization", "Cancer Management", "Interventional Oncology", and further identical keywords was done. Similar usage of MeSH keywords was observed in other datasets. The search was made as comprehensive as possible by double-checking the references of the included research ("reference checking"). Research citations were also evaluated ("citation tracing"). Furthermore, informal reports, letters to the editor, unpublished articles, and material shared on websites were removed from the list of downloaded files.

Results and discussion

IR Management (IRM)

IR is a highly specialised field of medicine that uses minimally invasive surgical methods and medical imaging to diagnose and treat diseases. Although the range of methods used by interventional radiologists is wide, all of these methods use medical imaging to guide minimally invasive surgery to minimise the risk to the patient [10-13].

Benefits of IR

IR has emerged as a viable approach for treating numerous disorders that previously necessitated invasive surgical procedures, now enabling a minimally invasive technique involving a small incision on the skin. One notable benefit of IR lies in its capacity to reach the profound anatomical structures of the human body through a minimally invasive approach involving a little incision, as well as the utilisation of diminutive needles and catheters. Implementing this technology mitigates the potential hazards posed to the patient and expedites the patient's recuperation process. Several advantages of utilising IR can be identified [14-16]. The potential benefits of pain treatment include reduced hospitalisation time, a decrease in the rate of infection, a lowered risk associated with surgery, and a shorter patient recovery period. IR aims to investigate methods for minimising intraoperative bleeding in surgical procedures, to reduce overall patient treatment expenses, and to minimise the utilisation of anaesthesia.

Numerous non-traumatic and traumatic bleeding issues are effectively managed by IR techniques. The concepts of embolisation and bleeding control can be applied to numerous anatomical locations and different kinds of traumatic injuries, even though practice guidelines have shed light on the function of IR techniques for specific injuries and disorders [17].

When medical or endoscopic treatment fails to stop gastrointestinal bleeding, using various embolic agents in transarterial embolisation may be considered a feasible option for surgical intervention. Transarterial embolisation is used to control the bleeding site in 40-80% of cases, with the success rate depending on the precise location of the bleeding [18,19]. The most common method used to identify the cause of non-variceal bleeding is upper or lower endoscopy. Patients with significant persistent bleeding of unknown cause that is assessed to be occurring at a rate of at least 0.5 mL/min may be treated with angiography. This diagnostic process makes it possible to locate the bleeding site, making it possible to carry out embolisation. CT angiography is frequently used to detect bleeding that exceeds a rate of 0.3-0.5 mL/min in people with lower levels of active haemorrhaging. Alternately, scintigraphy using red blood cells labelled with 99mTc can be used to identify the cause of slower bleeding, which happens at a rate of 0.05-0.1 mL/min. The next stage entails embolising the bleeding vessel after the diagnosis of active extravasation. Compared to patients who undergo surgical intervention, patients with transarterial embolisation typically show advanced age and a higher prevalence of medical comorbidities. However, compared to surgery, transarterial embolisation is associated with lower morbidity and similar survival rates [18,19]. However, compared to surgical procedures, transarterial embolisation has a higher re-bleeding rate. This phenomenon is commonly attributed to anomalies in the coagulation process, inaccurate angiographic localisation of the bleeding source, or insufficient embolisation.

The initial treatment for individuals with acute oesophageal variceal bleeding involves a combination of endoscopic gastroesophageal variceal ligation or sclerotherapy, together with pharmaceutical therapy [20]. The utilisation of transjugular intrahepatic portosystemic shunt (TIPSS) is warranted for patients who exhibit uncontrollable haemorrhage from oesophageal varices or experience recurrent bleeding after implementing pharmacological and endoscopic treatment methods [20]. The findings derived from randomised clinical studies indicate that TIPSS may exhibit greater efficacy compared to endoscopic and medicinal interventions in minimising recurrent variceal bleeding. However, it is important to note that this treatment approach does not appear to confer any advantage in terms of overall survival [21,22]. The administration of TIPSS within 72 hours of admission is correlated with reduced rates of treatment failure and mortality in comparison to a treatment regimen consisting of vasoactive medication, beta-blockers, endoscopic ligation, and subsequent TIPSS intervention [23]. The association between TIPSS and elevated rates of early hepatic encephalopathy has been documented in previous studies [21,22]. Other IR techniques that can be employed to manage variceal bleeding encompass balloon-occluded retrograde transvenous obliteration (BRTO) for gastric varices, transhepatic embolisation for gastroesophageal varices, portal vein recanalisation/thrombectomy with or without TIPSS, and partial splenic artery embolisation.

Types of Diagnostic and Treatment Methods in Intervention Imaging

Biopsy under imaging: US-guided biopsy procedures are often regarded as a highly precise approach for diagnosing various tumours and other abnormalities. This technique involves the insertion of a slender needle into the targeted tissue with the assistance of imaging modalities, facilitating the extraction of a sample or tissue tumours. The provided specimen will be forwarded to the pathology laboratory for further analysis. The utilisation of biopsy has superseded the practice of open surgery for sampling [24,25].

Many researchers have recorded their initial experiences performing biopsy procedures under the supervision of positron emission tomography (PET)/CT, using various techniques to improve the accuracy of needle location within the area demonstrating fluorodeoxyglucose (FDG) uptake [26-28]. As part of the approach, the combined PET/CT system's CT components are used to guide biopsy procedures. Using fused PET/CT images obtained utilising a single table position unenhanced CT and PET confirms needle insertion in the precise area, demonstrating FDG uptake [26-28].

With real-time PET/CT guidance, a newly developed electromagnetic needle tracking and navigation system enables an operator to complete biopsy procedures [29]. The suggested solution uses position sensors that are carefully placed on both the patient and the needle shaft, along with an ultralow electromagnetic field. Using US or CT guidance, this setup enables the system to precisely track the position and orientation of the biopsy needle in real time. The method also uses registration software to track the location of the needle tip and overlay this data onto cross-sectional images, like pre-procedural CT scans. Researchers claim that the spatial accuracy of this technology is sufficient for displaying picture-guiding information that is clinically significant throughout the biopsy process. By adopting this technology, procedures that would normally be deemed unfeasible by using traditional single-modality picture guidance can be carried out effectively [30-32].

Ureteral stenting and nephrostomy under imaging: In an individual with normal physiological functioning, urine transport involves the passage of urine from the kidney to the bladder via a slender and elongated conduit known as the ureter. The obstruction of the ureters and subsequent retrograde flow of urine to the kidney can be attributed to various medical conditions, including kidney stones, diverse tumours, infections, and blood clots. In this scenario, the medical practitioner employs a procedure wherein a stent or tube is introduced into the ureter using imaging techniques such as IR, intending to reinstate the regular urinary flow. Alternatively, if the approach mentioned above is unfeasible, nephrostomy is employed [33].

During the nephrostomy procedure, the patient assumes a prone position, and a catheter is inserted into the kidney through the posterior aspect of the patient's body. Typically, this tubular conduit serves to establish a connection between the kidney and a receptacle designed for the collecting of urine, facilitating the expulsion of accumulated urine from the renal system. The retention of the nephrostomy tube persists until the placement of a ureteral stent or the resolution of the obstruction in the ureter. To undergo a nephrostomy procedure, the patient must adhere to a fasting period of eight hours and seek medical advice before administering any medicine [34].

The use of imaging-guided retrograde ureteral access may lead to the development of a novel approach to the treatment of female urinary blockage. The performance of imaging-guided retrograde de novo ureteral access and double J (DJ) stent placement in women can be done without the requirement for cystoscopy while keeping a reasonable amount of radiation exposure. The scope of ureteric interventions performed within IR may be expanded by using this strategy. However, it is essential to evaluate patients using either cross-sectional imaging or ultrasonography to ascertain whether distorted trigonal architecture is brought on by the external compression of the bladder wall, as this has been identified as the main cause of technical challenges [35].

Evacuation and drainage of all types of cysts under imaging: Cysts in the abdomen, ovary, breast, liver, thyroid, etc. can be evacuated with IR using imaging techniques and with the help of special evacuation needles. To drain the cysts, the patient first receives local anaesthesia, and then, with the help of radiological images, intubation (catheterisation) begins to drain the cyst. This catheter is fixed on the skin with the help of a suture thread. After connecting the drainage bag to the catheter, drainage is performed. IR is also used to drain abdominal abscesses and infections. This method is also very effective in biliary drainage. In patients suffering from jaundice and itching due to the obstruction of the bile ducts, a special drainage catheter is inserted into the path of the obstruction with the guidance of radiological images. If the blockage is so severe that the catheter cannot pass through it, a bag is attached to the end of the tube to drain the bile out of the body [36].

CT offers enhanced characterisation of lesions concerning their spatial distribution, quantity, and dimensions. The identification of calcifications and the absence of contrast enhancement within an infiltrative mass may potentially suggest the diagnosis. CT remains the primary modality for detecting calcifications, which can serve as a valuable diagnostic feature, although they lack specificity [37]. MRI provides improved accuracy in evaluating the lesion, elucidating a diverse infiltrative hypovascular mass, including a mixture of solid and cystic tissue. T2-weighted imaging is often regarded as the sequence with the greatest specificity owing to its capacity to accurately visualise metacestode vesicles (microcystins) and regions of liquefaction necrosis (large cysts) as high signal intensities. On the other hand, the presence of granuloma tissue, coagulative necrosis, and calcification is observed as low signal intensities. The visual appearance of multivesicular lesions on T2-weighted imaging has been described in the literature as resembling either a cluster of grapes or a honeycomb structure [38].

Biliary interventions in IR: IR is becoming more and more important in the management of cancer patients at all stages, from early diagnosis to minimally invasive treatment of the tumour and any side effects that may result. Inflammatory processes, neoplastic growths, and infectious agents are just a few conditions that can cause the bile duct to become blocked or constricted. To treat biliary blockage or stenosis, IR typically implant biliary tubes in patients continuously treated for oncological diseases. Percutaneous transhepatic biliary drainage (PTBD) has become a critical part of treating biliary obstruction. Biliary drainage has been used as the main therapeutic technique and is widely recognised as an effective way to relieve malignant biliary obstruction [39].

Bile is a bodily fluid that is synthesised by the liver and plays a crucial role in the process of food digestion. The passage of bile occurs as it is transported from the liver through the bile ducts and ultimately into the intestines. In instances where an obstruction arises inside the bile duct, there is a potential for bile to retrograde into the liver, leading to the manifestation of various symptoms such as infection, nausea and vomiting, loss of appetite, elevated body temperature, yellowing of the skin and eyes (jaundice), as well as pruritus. The biliary drainage catheter facilitates the passage of bile from the liver into either a receptacle or the intestinal tract, contingent upon the specific type of catheter employed. The internal-external catheter is inserted percutaneously, traversing the dermal layers, and features both blockage-penetrating and side-hole structures [40]. This catheter facilitates the internal delivery of bile into the intestines and the exterior collection of bile into a receptacle. The catheter can be either capped or connected to a drainage bag. The external catheter is inserted percutaneously into the biliary ducts, positioned proximal to the site of obstruction, and consistently diverts the bile flow into a collection receptacle. An internal catheter refers to a medical device known as a stent that maintains the patency of a constricted region, allowing for the unobstructed flow of fluids into the intestinal tract. When employing an internal catheter, patients will not exhibit any discernible external bag or catheter [40].

Chorionic villus sampling (CVS): CVS is performed to detect genetic and congenital abnormalities during pregnancy. This test is performed in the 11th to 14th weeks of pregnancy under the guidance of US images. An anaesthetic is first injected into the mother to perform this test. Then, under US guidance, the sampling needle enters the uterine wall and extracts cell samples from the placenta. This method can diagnose diseases such as Down's syndrome, cystic fibrosis, and sickle cell anaemia [41].

Amniocentesis under US guidance: During pregnancy, the amniotic fluid surrounds and protects the fetus. There are various fetal cells and proteins in the amniotic fluid. Amniocentesis is a procedure in which, using IR, amniotic fluid is removed from the mother's uterus under US guidance. It is performed to detect chromosomal abnormalities during the fetal period and helps to diagnose genetic diseases such as Down's syndrome. This test is performed in women over 35 and women classified in the high-risk group during the first and second trimester screenings. The 14th week of pregnancy is the best time to perform amniocentesis, and no anaesthesia is needed. After examining the fetus's health with US, the sampling needle is inserted from the abdominal surface into the gestational sac, and about 20 cc of the fluid surrounding the fetus is drawn [42,43].

Angiography under imaging: In patients with chest pains, there is a possibility of blood vessel blockage and heart failure. Angiography is performed to diagnose vascular abnormalities such as blockage and aneurysm. It is performed under the guidance of imaging devices by inserting a very thin tube (catheter) through the groin or arm. In angiography, the contrast material enters the desired vessels through a catheter, and imaging is performed. This action is not a treatment process and only has diagnostic value. If the angiography results confirm the presence of blockage in the coronary arteries, the physician usually performs angioplasty [44].

Angioplasty and stenting in vessels: Angioplasty is performed to remove blockage and narrowing of coronary arteries. In angioplasty, under the guidance of medical imaging devices, a very thin tube is inserted through the groin or arm into the coronary arteries of the heart. The tip of this tube or catheter has an embedded balloon that can be inflated. When the catheter reaches the site of the narrowed artery, the balloon is inflated to compress the plaque against the artery wall. Usually, the balloon stays in the narrow place for about two minutes and then deflates. This process may be repeated several times at the doctor's discretion. Stenting is done to prevent re-occlusion of arteries. Stenting in vessels may be done separately or during angioplasty. Usually, following angioplasty and balloon deflation, a stent is placed inside the vein. In this process, the stent, as a barrier, keeps the vessel open and remains in it [45].

Application of Radiography in Medical Field and Radiology

Application of laser therapy: In general, a laser is used in the medical field in the three main areas of diagnosis, treatment, and beauty. In disease diagnosis, a laser identifies and evaluates musculoskeletal, orthopaedic, eye, dermatology, nerve, vascular, and oral and dental diseases. The use of lasers in the treatment of diseases can significantly reduce the amount of damage caused by surgery. The laser possesses the capability to incise the most resilient anatomical structures akin to a surgical scalpel, rendering it applicable in a wide array of surgical procedures. In laser surgery, wound healing is done better and faster, and less destruction occurs in the treatment area. The application of laser in treating skin diseases and beauty has gained a special place with the arrival of new and advanced devices. The laser heats up and eventually destroys some sensitive tissue molecules. Considering that the laser wavelength is known, it affects only certain structures. The laser is utilized for the treatment of various dermatological conditions such as skin disorders, lesions, moles, excessive hair growth, wrinkles, and scar removal. In order to effectively treat each individual instance, it is imperative to utilize a laser wavelength that is specifically tailored to the unique characteristics and requirements of the situation at hand [46,47].

Radiofrequency (RF) in IR: RF is a low-risk treatment method that can destroy tumours of the liver, lung, bone, breast, etc. The high temperature caused by these waves destroys cancer cells in the target organ. RF is performed under the guidance of imaging devices and without hospitalisation and anaesthesia. The RF treatment is particularly well-suited for patients who are unable to undertake surgical procedures due to their overall health status or the specific location of the tumour [48,49].

Embolisation in IR: Embolisation or embolotherapy is a semi-invasive method to block blood vessels. This method is used to stop or prevent bleeding. Disabling structures such as tumours is also one of the uses of embolisation, which reduces the blood supply to the tissue. In this method, objects or materials are introduced into the blood supply system under the guidance of imaging devices to block the desired vessel. These objects and materials include items such as coils, ethanol, microspheres (such as polyvinyl alcohol (PVA)), gelatin sponges (gel foam), etc. Embolisation plays a role in treating diseases such as aneurysms, vascular anomalies, lymphatic malformations, haemangiomas, pulmonary artery fistulas, arteriovenous malformations, and bleeding caused by trauma [50,51].

Application of IR for Cancer Management

Improvements in IR during the past decade have occurred in conjunction with similar developments in diagnostic imaging. Most lesions can be identified using diagnostic imaging, but a definitive diagnosis and course of therapy rely on the pathological examination of the cancerous tissue. Since medications with specific molecular targets are gaining much interest, tissue analysis will become more important when deciding on therapy [52]. IR greatly helps in tissue sample collection for analysis. One of the most frequent procedures by interventional radiologists is imaging-guided biopsy. A few conditions inhibit image-guided biopsy from being used. Since core biopsy removes more tissue than small needle aspiration, it is preferable because it preserves the tumour's cellular form and structures [53].

Using a dual-system biopsy in doing image-guided core biopsies is common practice. A dual-system biopsy collects tissue samples with the central biopsy equipment incorporated via a guiding needle on a detachable rod. Multiaxial systems, commonly used for biopsies, have a spring-loaded mechanism and range in gauge from 16 to 20. The multiaxial method allows for collecting many samples with a single insertion needle. If required, the needle can even be used again for fine needle aspiration [54].

Depending on the site of the lesion, image-guided biopsies may be conducted with the use of US, CT, MRI, or X-rays. US guidance is effective and safe because it provides real-time images of the biopsy instrument and lesion. When the US cannot detect a lesion, CT can be employed. Biopsies guided by an MRI need special tools and are often done on soft tissue [55].

Long-term intravenous access is necessary for most malignancies, requiring regular blood testing, contrast injections, and chemotherapy. Vein access is crucial for the majority of interventional procedures. Interventional radiologists are trained to insert permanent venous access devices, including central venous catheters and subcutaneous central venous ports. Several internists can perform central venous access uncommonly, such as translumbarly, transhepatically, and through the azygos systems [39].

Most IR treatments, such as central venous access and biopsies, need conscious anaesthetic overseen by IR nursing staff. Due to their low risk and low complication rate, these operations can be performed on an outpatient basis. Interventional radiologists are increasingly playing a role in identifying and managing cancer patients, leading to an expanding subspecialty of interventional radiologists known as "oncologist internists". Recently, interventional oncology (IO) has been called the fourth pillar of oncology, along with radiation, surgical and clinical oncologists. Interventional radiologists are, in some cases, becoming providers of services to patients with certain cancers and a slight shift toward minimally invasive treatments [56,57].

IO: Oncology is a branch of medicine that studies tumours and cancers. A doctor specialising in this field is called an oncologist. Minimally invasive image-guided treatments with comparable clinical outcomes to surgery have contributed significantly to IO's explosive expansion. Managing oncology patients effectively necessitates familiarity with current therapy options. Interventional oncologists routinely perform image-guided ablation of solid organ malignancies. Ablation can be performed using several different energy sources, such as RF, microwave, or cryoablation. Ablation is a common method for treating organ tumours like the liver, kidney, and lung. Also, it is typically used for treating smaller tumours and works quite well [58,55]. Catheter-based therapies are frequently used for the treatment of liver cancers, both primary and metastatic. These therapies rely on liver tumours receiving both artery and vena cava blood, primarily supplying the normal liver [58].

## Conclusions

The present narrative review highlights the significance of IR in acquiring a tissue sample for tissue analysis. Another typical procedure for interventional radiologists is imaging-guided biopsies. Among the different biopsy methods, core biopsy is preferable because larger tissue volumes are removed compared to aspiration with a fine needle. Therefore, the cell morphology and overall structure of the tumour are preserved.

Despite being formed as a separate specialty only 50 years ago, the discipline of IR has experienced a remarkable expansion in the scope and importance of its services. Interventional radiologists have evolved over the past three to four decades from largely operating in reading rooms to taking on a more clinical role, providing both consultation and procedural abilities. Various minimally invasive techniques are included in IR, which has developed from a diagnostic modality to a therapeutic strategy. These operations include fluid/abscess drainage, oncologic medications, and vascular interventions. The morbidity that patients in both inpatient and outpatient settings experienced has been significantly reduced because of these interventions, which is significant.
